# Snow-cover loss attenuates the effects of N addition on desert nutrient cycling and microbial community

**DOI:** 10.3389/fpls.2023.1166897

**Published:** 2023-07-20

**Authors:** Yaru Yang, Weiguo Liu, Jonathan M. Adams, Bin Song

**Affiliations:** ^1^ College of Ecology and Environment, Xinjiang University, Urumqi, China; ^2^ Key Laboratory of Oasis Ecology of Education Ministry, Xinjiang University, Urumqi, China; ^3^ Xinjiang Jinghe Observation and Research Station of Temperate Desert Ecosystem, Ministry of Education, Urumqi, China; ^4^ School of Geography and Ocean Science, Nanjing University, Nanjing, China

**Keywords:** bacteria, biological soil crusts, desert, enzyme, nitrogen, snow

## Abstract

Desert ecosystems are sensitive to nitrogen (N) deposition. Considering snow is an important source of soil water, which is vital for plant growth and the biogeochemical cycle in desert areas. The effects of N deposition on biological soil crusts (BSCs) could be impacted by the removal of snow-cover. Here, we established a split-plot experiment in the Gurbantunggut Desert to examine the effects of snow-cover treatments on soil nutrients, enzyme activities, and the bacterial community under various N addition. The removal of snow-cover reduced the soil nutrients with light and moderate N addition, it also reduced the activities of urease (URE) and alkaline phosphatase (PHOS). The structural equation model (SEM) result indicated that low soil moisture (SMO) under snow-uncover inhibited the bacterial community, particularly suppressed bacterial diversity. Additionally, N addition indirectly affected the bacterial community *via* modifications to soil nutrients, and soil organic matter (SOM) (*P* < 0.001) was the crucial factor. Snow-uncover weakened soil nutrient and enzyme responses to N addition, indicating that snow-cover removal reduced the sensitivity of the desert ecosystem to N deposition. The study highlights the critical role of snow-cover in the desert ecosystem, raising our awareness of the ecological risks of BSCs in future global change.

## 1 Introduction

Global change is accelerated by human activities, particularly precipitation patterns and atmospheric nitrogen (N) deposition, which have an impact on the ecological functioning of terrestrial ecosystems, including soil nutrient dynamics (Zhang et al., 2020), greenhouse gas fluxes (Chae et al., 2016; Liu et al., 2017), and soil microbes (Ma et al., 2020). The precipitation in the desert is decreasing due to global climate change (Epps et al., 2006; Li et al., 2016), affecting the local typical vegetation biological soil crusts (BSCs) (Zhao et al., 2018). BSCs, composed of bacteria, fungi, algae, lichens, and mosses (Blay et al., 2017), alter the nutrient cycle and gas exchange in ecosystems as well as their functionality (Liu et al., 2017). And, microorganisms are essential to the BSCs physiological properties and nutrient cycles (Maier et al., 2018). N supply is considered a key regulator of soil microbial activity (Wang et al., 2023), while precipitation is known as a major regulator of the growth of BSCs (Wertin et al., 2012; Wu et al., 2015), and is also described as having the capacity to affect microbial communities and enzyme activities (Hu et al., 2020). There are only a few studies on the response of desert soil microbial communities to snow, but this response may be a key factor for soil nutrient changes in winter (Yan et al., 2018).

BSCs are major contributors of N for desert ecosystems (Qi et al., 2022), and more often than not, they are mentioned for their impact on N cycling (Bowker et al., 2008; Wang et al., 2021a). Dryland ecosystems are usually N-limited (Lu et al., 2022), and N cycling is influenced by low soil organic matter, high soil pH, extreme water potential, and temperature (Sinsabaugh et al., 2015). Correspondingly, changes in soil properties caused by the N addition, such as pH, electrical conductivity, and ammonium content can indirectly affect the composition of BSCs (Rong et al., 2022). In addition to reducing bacterial diversity, long-term N addition also modifies soil enzyme activity, bacterial community structure, and bacterial function (Sinsabaugh et al., 2015). Wang et al. (2015) came to the conclusion that cyanobacteria predominate during moderate N addition, while proteobacteria and actinobacteria predominate during excessive N. Overall, N addition affects the BSCs, while the response of the BSCs to N deposition in the Central Asian desert is considered to be dependent on variations in precipitation (Cui et al., 2017).

Winter snowfall influences the establishment and development of BSCs in temperate desert regions of China (Zhao et al., 2016a), which has a significant impact on the dynamics of soil nutrients and microbial biomass (Zhao et al., 2018), and provides conditions for their survival in hostile situations (Hui et al., 2016). Higher soil moisture under snow-cover promotes net N mineralization, which in turn increases soil N availability and alleviates N limitation of the soil microbial community (Zhang et al., 2015; Chen et al., 2023). The increased snow-cover alters the structural composition and functional communities of BSCs by increasing the content of soluble proteins and photosynthetic pigments (Hui et al., 2018). Meanwhile, the ecological niche of cyanobacteria, which are essential photosynthetic organisms in BSCs, is also impacted by soil qualities that are changed by snow accumulation and irradiance (Zhang et al., 2021a). Several studies have found that plants and soil microorganisms in desert ecosystems are sensitive to changes in precipitation and N deposition (Vishnevetsky and Steinberger, 1996; She et al., 2018). Hui et al. (2022) also demonstrated that variations in snow depth have a negative impact on the availability of carbon and nutrients as well as microbial biomass. These fluctuations may also impair the structure and functionality of BSCs communities in arid regions.

The Gurbantunggut Desert has a large distribution of BSCs and more potential sources of N from agricultural and industrial activities (Fan et al., 2013; Huang et al., 2021), yet studies on the effects of snow-cover and N addition on nutrient cycling and microbial communities of BSCs are lacking. Here, we performed a split-plot experiment with snow-cover and N addition to examine the following questions: (1) how snow-cover altered the nutrient status of BSCs under different N additions, (2) how are soil enzyme activities related to N acquisition in BSCs response to snow-cover under different N additions, (3) effects of snow-cover on bacterial structure and function under different N addition levels, and (4) address the underlying mechanisms that drive soil nutrient dynamics and microbial communities.

## 2 Materials and methods

### 2.1 Sample sites

The experimental site was located at the southern edge of the Gurbantunggut Desert (44°11′–46°20′N, 84°31′–90°00′E), which has an area of about 48,888 km^2^ and is near the Manas National Wetland Park (Hui et al., 2022). The site lies in the Northern Tianshan Economic Zone with an annual precipitation of 79.5 mm and an average temperature (T) of 7.26°C (Wu et al., 2006). Gurbantunggut has good survival and a strong competitive place for BSCs with alkaline soil (pH 8.69–8.89) (Zhang et al., 2022a), which is adjacent to the industrial zone with high N deposition (44°30′N, 87°91′E). Moreover, it is an excellent winter pasture with more than a hundred species of plants, and the flora is in transition from Central Asia to the Central Asian desert. The desert flora of central Asia is dominant in the western and central parts of the desert, and ephemeral plants are widely distributed. Snow-cover (6–16 cm) is during winter (Hui et al., 2022), mainly from November to March (Stable snow period: 100-150 days) (Zhang et al., 2022b). The vegetation in China’s largest fixed and semi-fixed desert is dominated by *Haloxylon ammolondren*, *Haloxylon persicum*, *Ephedra distachya* and *Ceratoides latens*, with well-developed BSCs widely dispersed, moss crusts distributed under the plant canopy and in plant gaps, some algal crusts distributed on the bare ground, and only a few lichens distributed (Huang et al., 2021).

### 2.2 Experimental design

Considering the distribution of moss crust, three experimental plots (100 m × 100 m) were randomly selected at the study area, and six samples (10 m × 10 m) were randomly selected in each plot for snow and N treatment. The distance between each sample area was greater than 20 m. Besides, the split-plot experiment involved two snow treatments in main plots: (1) snow-cover (natural snow cover) (S), and (2) Snow-uncover (covered with tarpaulin before snowfall and removed after snowfall) (UnS). According to the 3.6 g N·m^−2^·year^−1^ atmospheric N deposition during the past decade (Huang et al., 2015), each application with a three-level N addition of ^15^NH_4_^15^NO_3_ solution (dissolved in 100 ml non-ionized water) in sub-plots, which was added and sprayed evenly on the sample square with a sprinkler on November 1, 2018 (Huang et al., 2021): (1) high N (7.2 g N·m^−2^·year^−1^) (H), (2) moderate N (3.6 g N·m^−2^·year^−1^) (M), (3) light N (1.8 g N·m^−2^·year^−1^) (L), and each application had three replicate plots. A portion of the collected soil samples was quickly packed on dry ice, brought back to the laboratory, and kept in a freezer (−80°C) within 12 h. The other portion of each uniform sample was divided into several parts and air–dried for analysis of soil nutrients and enzymes. Soil temperature (T) (°C) in the top 0–10 cm layer of the soil near the collar was measured with an auxiliary sensor attached to the LI-COR 8150. Soil moisture (SMO) (%) was measured after drying in an oven at 105°C for 24h (Wu et al., 2013).

### 2.3 Soil sample collection

The soil was collected on March 30, 2019, when snowfall stopped. Three random soil cores with a depth of 5 cm and a diameter of 3.5 cm were collected with a sterile shovel and then combined into one soil sample to reduce geographical differences. Finally, 18 soil samples were collected and soil temperature was measured (2 snow treatments × 3 N levels × 3 repetitions). The soil samples were placed in a portable freezer, and the roots and stones of plants were removed with a 2 mm screen, and then transferred to the laboratory.

### 2.4 Physicochemical analysis of soil sample

Soil organic matter (SOM) was determined by the loss-on-ignition method after ash at 550°C for 4 h (Wu et al., 2013). Total nitrogen (TN) was determined following the Kjeldahl digestion method on a nitrogen analyzer system (KJELTEC 2300 AUTO SYSTEM II, Foss Tecator AB, Höganäs, Sweden) (Gallaher et al., 1976). Soil pH was determined in a 1:10 (w:v) soil-distilled water suspension with a pH meter (HQ30d, Hach. USA) in suspension (dry sediment/water, 1:5). To obtain the extraction solution to measure soil ammonium (NH_4_^+^-N) and nitrate (NO_3_^−^-N), 20 g of fresh soil from each sample was taken and 100 ml KCl (2 mmol L^-1^) was added, and the mixture was shaken and filtered (Edwards and Jefferies, 2013). The values of available nitrogen (AN) were measured using alkaline KMnO_4_ (Hui et al., 2022).

Urease (URE) was measured by urea and expressed as mg NH_4_-N g^−1^ soil 24 h^−1^. Alkaline phosphatase (PHOS) was measured according to the method described and expressed as mg phenol g^−1^ 3 h^−1^ (Wei et al., 2017). To measure alkaline protease (AprX), 1 g soil was sealed in the 2.5 mL Tris buffer (0.2 M, pH 8.0) and Na-caseinate solution (2%) mixture. After 2 h of water bath (50°C), 5 mL trichloroacetic acid (10%) was added to continue precipitation. Subsequently, 0.5 mL of the solution was mixed with 1 ml Na_2_CO_3_ (14 M) and 0.2 mL Folin-Ciocalteu reagent (three-fold diluted) and then incubated in an Eppendorf tube (2 mL) for 5 min, centrifuged for 1 min (~16,400×*g*), and the tyrosine concentration was measured by colorimetry 680 nm (Tian and Shi, 2014). Polyphenol oxidase (PPO) was measured by the amount of purpurogallin and expressed as mg purpurogallin g^−1^ soil 2 h^−1^. Moreover, 5 g of soil was added to sucrose solution and incubated for 24 h at 37°C, and invertase was measured by mg glucose equivalent g^−1^ soil 24 h^−1^. Peroxidase (POD) was measured with 9 g soil agitated in 25 mL 0.2 M phosphate buffer (pH 6.0) for 5 min and centrifuged at 8000 × g for 10 min. The supernatant (2.7 mL in a spectrophotometric cuvette) was filtered using 0.22 µm Durapore filters (Millipore) for sterilization and added 0.3 mL 0.06% H_2_O_2_ in 0.05 M phosphate buffer (pH 6.0) and 0.05 mL 0.5% 0-dianisidine in methanol (Serra-Wittling et al., 1995). Nitrate reductase (NR) was measured by diaminodiphenyl sulfone based on nitrate determination (Daniel and Curran, 1981). Cellulase was estimated by 3,5-dinitrosalicylic acid using carboxymethyl cellulase (Das and Mondal, 2019).

### 2.5 Extraction and sequencing of DNA

Firstly, the extracted genome DNA of the samples were detected by the purity and the concentration of 1% agarose gel. DNA was diluted to 1 ng μL^-1^ in sterile water as a template. Specific barcode primers were used according to the selection of sequencing regions (V4). 515F: GTGCCAGCMGCCGCGGTAA and 907R: CCGTCAATTCCTTTGAGTTT (Xu et al., 2021). Phusion^®^ High-Fidelity PCR Master Mix (New England Biolabs) was used for all PCR reactions. Secondly, the PCR reactions were mixed with 1×loading buffer (contained SYB green) in equal amounts according to their concentrations, purified by 2% agarose gel electrophoresis, and the target bands were recovered by shearing. GeneJET™ Gel Extraction Kit (Thermo Scientific) was used for product purification. Finally, the Ion Plus Fragment Library Kit 48 rxns (Thermo Scientific) was used to generate the sequencing library. After the generated library passed Qubit quantification and library detection, Ion S5™XL was used for on-machine sequencing. IonS5™XL data was exported for offline analysis.

### 2.6 Metagenomic analysis

Cutadapt (v1.9.1, http://cutadapt.readthedocs.io/en/stable/) was used to filter low-quality data. Raw reads were obtained by preliminary quality control, and chimeric sequences were then removed (Rognes et al., 2016). By comparing the reference database (Silva database, https://www.arb-silva.de/), reading and then removing the chimeric sequences, the final data (clean reads) was obtained. Uparse software (v7.0.1001, http://www.drive5.com/uparse/) was used to cluster clean reads of all the samples. Sequences were clustered into operational taxonomic units (OTUs) with 97% identity. Species annotation was performed for OTU sequences, and species annotation analysis was performed using the Mothur algorithm and the SSU rRNA database SILVA132 (http://www.arb-silva.de/) (Liu et al., 2020a). MUSCLE (v3.8.31, http://www.drive5.com/muscle/) was used for rapid multiple sequence alignment of all OTU sequences. In the end, using the bioinformatics software package PICRUSt, metagenomic function prediction based on the KEGG database was performed on 16S rRNA (marker gene) sequence data (Langille et al., 2013).

### 2.7 Data analysis

Box plots were created in R (v4.0.2) to show the effects of snow-uncover on soil environmental factors, bacterial communities, and enzyme activities under different N addition levels. Two-way ANOVA was used to determine the effects of N addition and snow-uncover on soil environmental factors and enzyme activities by SPSS19. In addition, R (v4.0.2) was used to perform Heatmap, redundancy analysis (RDA) and LDA to compare the relationship among the enzyme activities, environmental factors, and bacterial abundance, and reveal the functional differences in microbial communities. Cytoscape v3.8.2 was used to analyze the effect of bacterial communities with snow-cover within the network. A structural equation model (SEM) was performed in R (v4.0.2) to analyze the effect of snow-uncover on desert BSCs.

## 3 Results

### 3.1 Effects of snow-cover on soil environmental factors across different N addition levels

**Figure 1:**
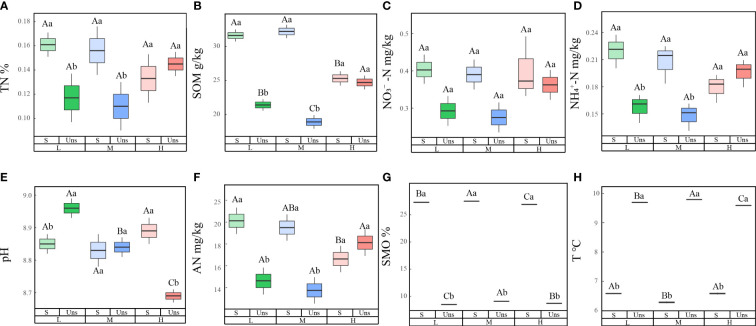
Effects of nitrogen addition and snow-cover treatments on soil environmental factors. Three levels of N addition (L = 1.8 g N·m^−2^·year^−1^, M = 3.6 g N·m^−2^·year^−1^, H = 7.2 g N·m^−2^·year^−1^). Total nitrogen (TN), soil organic matters (SOM), soil moisture (SMO), pH, temperature (T), available nitrogen (AN), ammonium (NH_4_^+^-N), nitrate (NO_3_^−^-N). Snow-cover (S), and snow-uncover (Uns). **(A–H)** indicate the TN, SOM, NO_3^−^_-N, NH_4^+^_-N, pH, AN, SMO, and T of environmental factors, respectively. Different lowercase and capital letters denote significant differences (*P* < 0.05) between treatments of nitrogen addition and snow-cover treatments, respectively. Error bars represent the standard error (SE) (*n* = 3).

In contrast to soil nutrients under snow-cover, snow-uncover caused nutrients to increase with greater N additions. Multiple analyses showed lower concentrations of TN (*P* < 0.001), SOM, AN (*P* < 0.001), NO_3_^−^-N (*P* < 0.05), and NH_4_^+^-N (*P* < 0.001) with snow-uncover than snow-cover until the highest N was added, indicating snow-uncover diminished the nutrient storage capacity of soils. In addition, pH (*P* < 0.01) with snow-uncover presented higher values under light N addition, and the pH with different additions of N showed a greater difference than the samples with snow-cover (**Figure 1**; **Table 1**), indicating that snow-cover reduced the soil acidification caused by N addition and remained stable. With increasing N addition, soil TN, NH_4_^+^-N (*P* < 0.01), NO_3_^−^-N and AN (*P* < 0.001) decreased with snow-cover but increased with snow-uncover. Likewise, SOM (*P* < 0.001) with snow-uncover decreased with increasing N addition levels and increased by 76.5% with moderate to high N addition. Besides, SOM (*P* < 0.001) with snow-cover decreased with increasing N addition levels and significantly declined with high N addition. In summary, the interaction between snow and N significantly changed the soil environmental factors and the underlying causes deserve further analysis and discussion (**Table 1**).

**Table 1:** Effect of nitrogen addition, snow-cover treatments, and their interaction on soil environmental factors, as indicated by two-way ANOVA statistics.

	TN	SOM	SMO	pH	T	AN	NH_4_^+^-N	NO_3_^−^-N
N	4.32	249.1590 ***	3600.00 ***	302.25 ***	900.00 ***	8247.01 ***	609.10 **	1.66
Snow	91.26 ***	726012.50 ***	3541338.46 ***	24.00 **	307200.00 ***	377909.77 ***	35.81 ***	15.56 *
N × snow	48.78 ***	164412.50 ***	323.08 ***	281.63 ***	700.00 ***	201607.73 ***	331.63 ***	1.319

Values represent F-values. Total nitrogen (TN), soil organic matters (SOM), soil moist (SMO), pH, soil temperature (T), available nitrogen (AN), ammonium (NH_4_^+^-N), nitrate (NO_3_^−^-N). Snow-cover (S), and snow-uncover (Uns). *P < 0.05; **P < 0.01; ***P < 0.001 (Pr > F).

### 3.2 Effects of snow-cover on enzyme activities across different N addition levels

**Figure 2:**
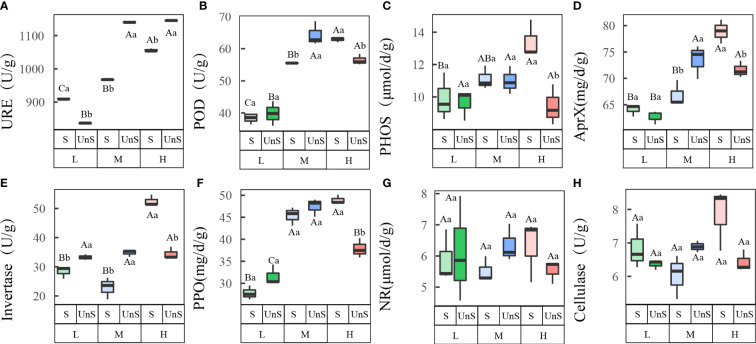
Effects of snow-cover treatments and nitrogen addition on soil enzyme activities. Three levels of N addition (L = 1.8 g N·m^−2^·year^−1^, M = 3.6 g N·m^−2^·year^−1^, H = 7.2 g N·m^−2^·year^−1^). Urease (URE), peroxidase (POD), alkaline phosphatase (PHOS), alkaline protease (AprX), invertase, polyphenol oxidase (PPO), nitrate reductase (NR), cellulase. Snow-cover (S), and snow-uncover (Uns). **(A–H)** indicate the soil enzyme activities of URE, POD, PHOS, AprX, invertase, PPO, NR, and cellulase, respectively. Different lowercase and capital letters denote significant differences (*P* < 0.05) between treatments of nitrogen addition and snow-cover treatments, respectively. Error bars represent the standard error (SE) (*n* = 3).

Most soil enzyme activities showed significant changes when N was added, and snow-uncover slowed the response of most enzyme activities to N addition (**Figure 2**; **Table 2**). With the increasing N addition levels, the activities of URE (*P* < 0.001), POD (*P* < 0.001), PHOS, AprX (*P* < 0.01), and PPO (*P* < 0.001) rose with snow-cover and showed a peaking trend with snow-uncover, while invertase (*P* < 0.01), NR, and cellulase showed a valley trend with snow-cover and a peaking trend with snow-uncover. Furthermore, at each N supplemental level, URE and invertase indicated that snow-cover induced significant changes, whereas POD, PHOS, PPO, and AprX did not show significant changes until reaching a high N concentration. In addition, the combination of N and snow-cover showed significant interaction with URE (*P* < 0.001), PHOS (*P* < 0.01), invertase (*P* < 0.001), PPO (*P* < 0.001), POD (*P* < 0.001), AprX (*P* < 0.001) and cellulase (*P* < 0.01) (**Table 2**).

**Table 2:** Effect of nitrogen addition, snow-cover treatments, and their interaction on soil enzyme activities, as indicated by two-way ANOVA statistics.

	URE	PHOS	Invertase	PPO	POD	AprX	NR	Cellulase
N	16009.30 ***	3.92	61.81 **	119.18 ***	115.03 ***	76.16 **	0.01	1.75
Snow	2210.43 ***	6.75 *	0.20	3.03	0.97	0.34	0.12	2.07
N × snow	2929.63 ***	5.08	76.59 ***	22.34 **	12.48 **	11.74 **	2.64	7.60 *

Values represent F-values. Urease (URE), alkaline phosphatase (PHOS), invertase, polyphenol oxidase (PPO), peroxidase (POD), alkaline protease (AprX), nitrate reductase (NR), cellulase. *P < 0.05; **P < 0.01; ***P < 0.001 (Pr > F).

### 3.3 Effects of snow-cover on soil bacterial community composition and functions under different N addition levels

**Figure 3:**
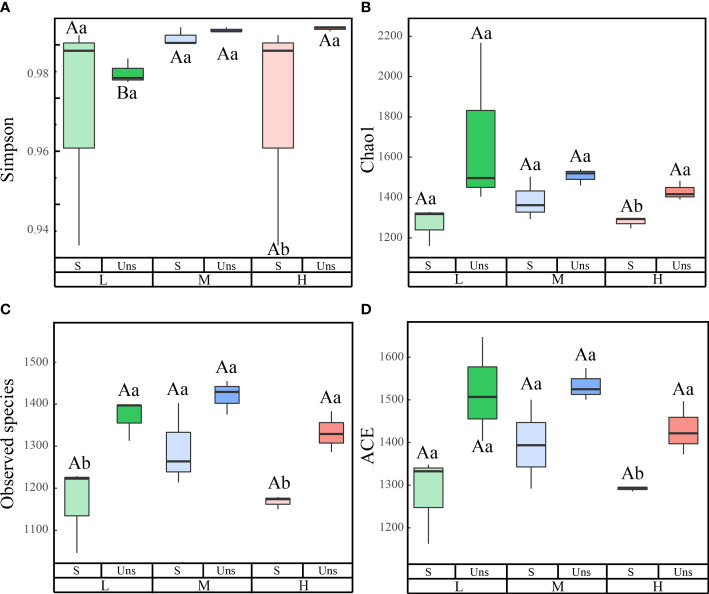
Effects of snow-cover treatments and nitrogen addition on bacterial diversity index. L = 1.8 g N·m^−2^·year^−1^, M = 3.6 g N·m^−2^·year^−1^, H = 7.2 g N·m^−2^·year^−1^. Snow-cover (S), and snow-uncover (Uns). **(A–D)** indicate the simpson, Chao1, observed speciese, and ACE of the bacterial community, respectively. Different lowercase and capital letters denote significant differences (*P* < 0.05) between treatments of nitrogen addition and snow-cover treatments, respectively. Error bars represent the standard error (SE) (*n* = 3).

**Figure 4:**
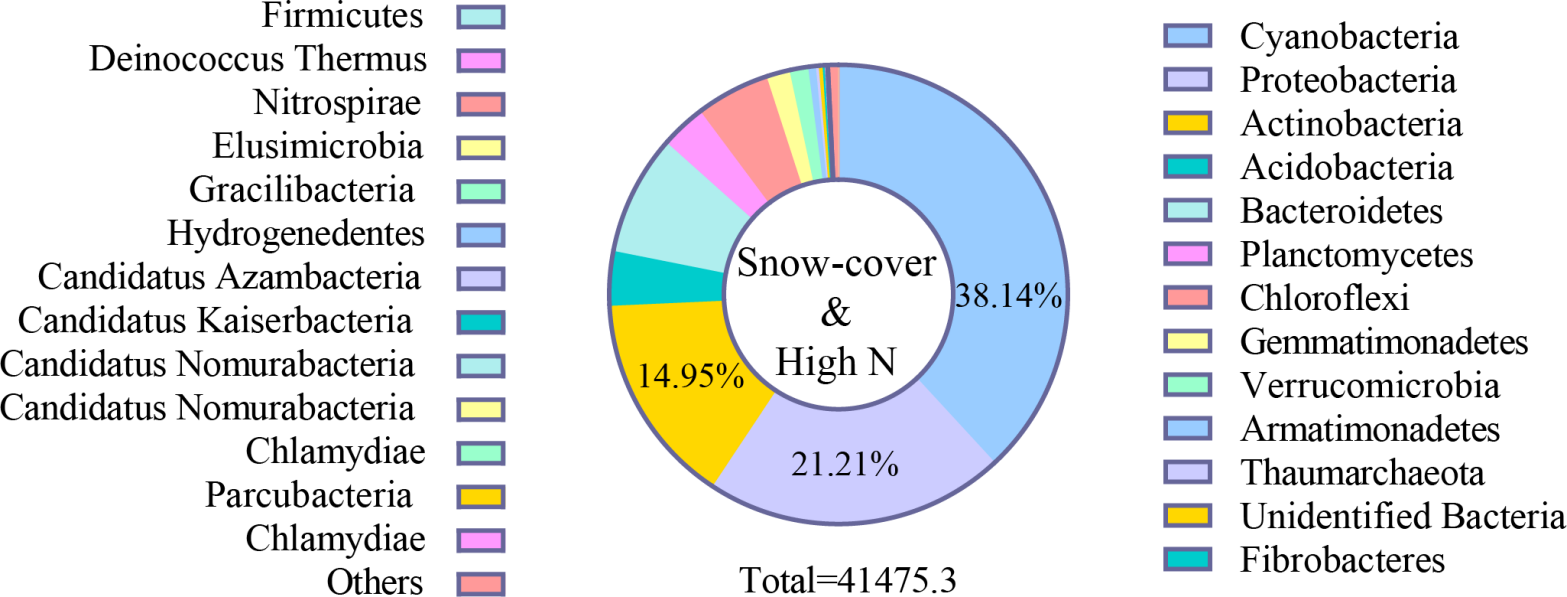
Relative abundance of bacteria at phylum level with snow-cover treatments and high nitrogen addition. Different colors represent bacteria in the different phyla.

**Figure 5:**
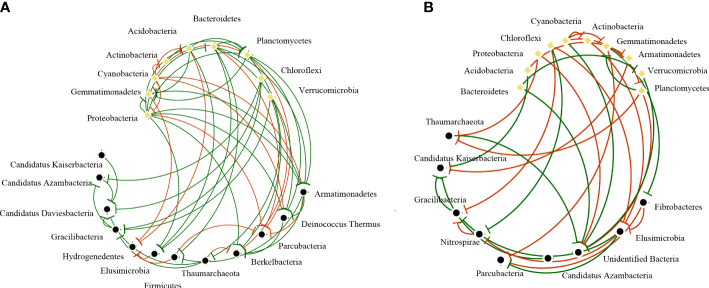
Network analysis among bacterial communities with snow-cover **(A)** and snow-uncover **(B)**. Nodes represent the various bacteria phyla, and the yellow dots represent core bacteria (Relative abundance > 1%). The red line indicates negative interaction, whereas the green line indicates positive interaction (|R| > 0.6, *P* < 0.05).

In all samples, snow-uncover was observed to exacerbate the response of bacterial community abundance and diversity to N addition at the phylum level (**Figure 3**; **Table 3**). Snow-uncover significantly increased the number of phyla in bacterial communities with light and high N addition. For high N addition, the snow-uncovered bacterial community significantly presented lower diversity and higher abundance (**Figure 3**). At the same time, cyanobacteria were the dominant phylum with snow-cover and high N addition (**Figure 4**), whereas proteobacteria were dominant in the other status. Moreover, association networks were generated to characterize the co-occurrence in the bacterial community in the two snow treatments (**Figure 5**). A total of 26 nodes and 81 edges were on the left, whereas 27 nodes and 57 edges were on the right of the figure, indicating bacterial interaction changed by snow-uncover. Cyanobacteria, proteobacteria, acidobacteria, gemmatimonadetes, bacteroidetes, planctomycetes, chloroflexi, and verrucomicrobia were presented as the core bacteria with the application of both snow-cover and snow-uncover. The remarkable divergence was that armatimonadetes were the core bacteria only when snow was removed. In addition, the module on the left showed a positive correlation and higher intensity correlation among the bacteria with snow-cover (A). While the module on the right showed a more negative correlation and sparse correlation among the bacteria with snow-uncover (B), indicating weak connectivity with snow-uncover.

**Table 3:** Effect of nitrogen addition, snow-cover treatments, and their interaction on bacterial diversity, as indicated by two-way ANOVA statistics.

	Simpson	Observed species	ACE	Chao1
N	1.74	2.23	1.24	0.49
Snow	1.87	61.64 ***	40.98 **	0.97 *
N × snow	0.48	1.12	1.55	1.66

Values represent F-values. *P < 0.05; **P < 0.01; ***P < 0.001 (Pr > F).

**Figure 6:**
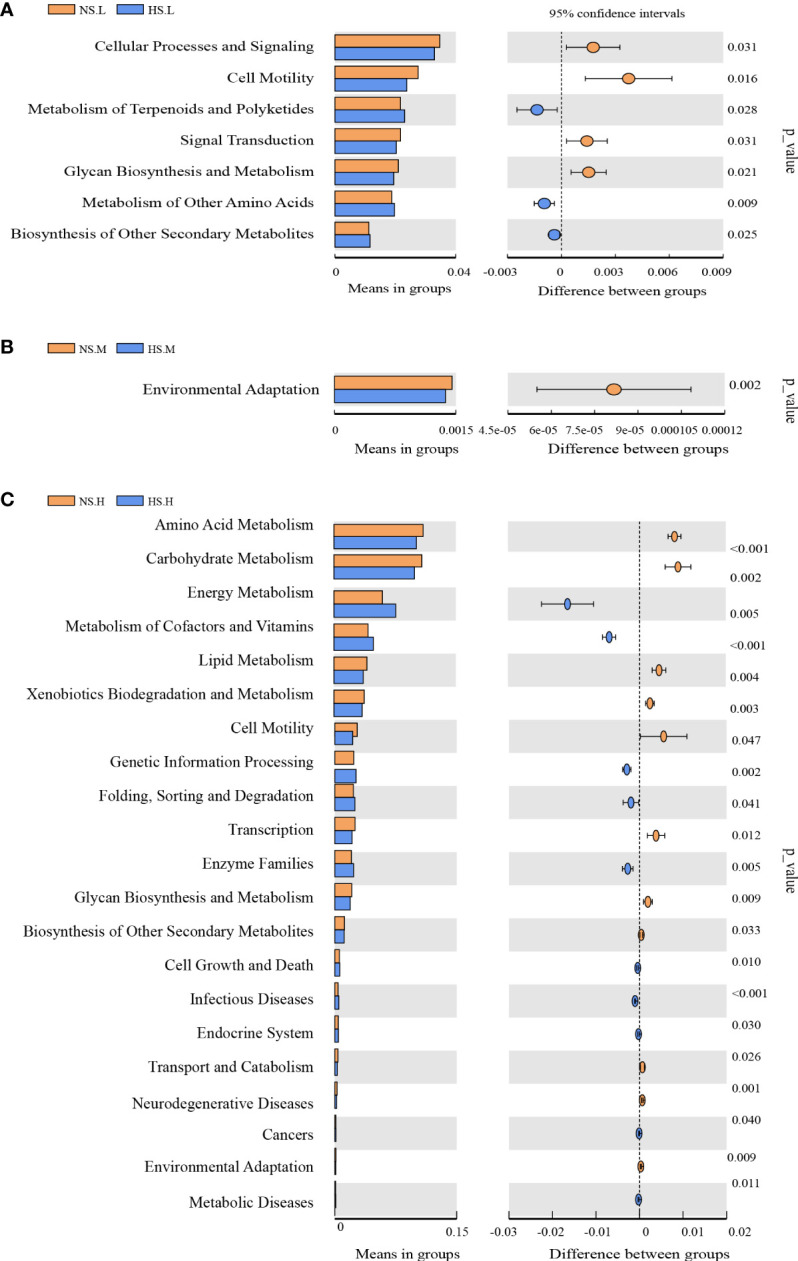
Effects of snow-cover treatments and nitrogen addition on the function of the microorganism. Light N addition **(A)**, Moderate N addition **(B)**, High N addition **(C)**. NS.L (Snow-uncover and light nitrogen), HS.L (Snow-cover and light nitrogen), NS.M (Snow-uncover and moderate nitrogen), HS.M (Snow-cover and moderate nitrogen), NS.H (Snow-uncover and high nitrogen), HS.H (Snow-cover and high nitrogen). Lineages with LDA values higher than 3.5 were displayed. Different colors represent different snow-cover treatments (Orange: Snow-uncover, Blue: snow-cover). Extended error bar plots indicate significantly different predicted functional categories. *p*-values (95%).

A significant discrepancy between the T-tests was found in the metabolism function of the BSCs bacterial community under the three N addition levels (**Figure 6**). After adding light N, the snow-uncovered bacterial community showed stronger glycan metabolic function, whereas the snow-covered bacterial community showed stronger terpenoids, amino acid metabolism, and other secondary metabolites biosynthesis function. However, with the high N, the snow-uncovered bacterial community showed stronger amino acid, carbohydrate, lipid, chitosan, and biosynthesis of heterogeneous biological metabolism, whereas the snow-covered bacterial community showed higher energy, auxiliary factor, vitamin and stronger enzyme metabolism function.

### 3.4 Relationships among environmental factors, bacterial community and enzyme activities

**Figure 7:**
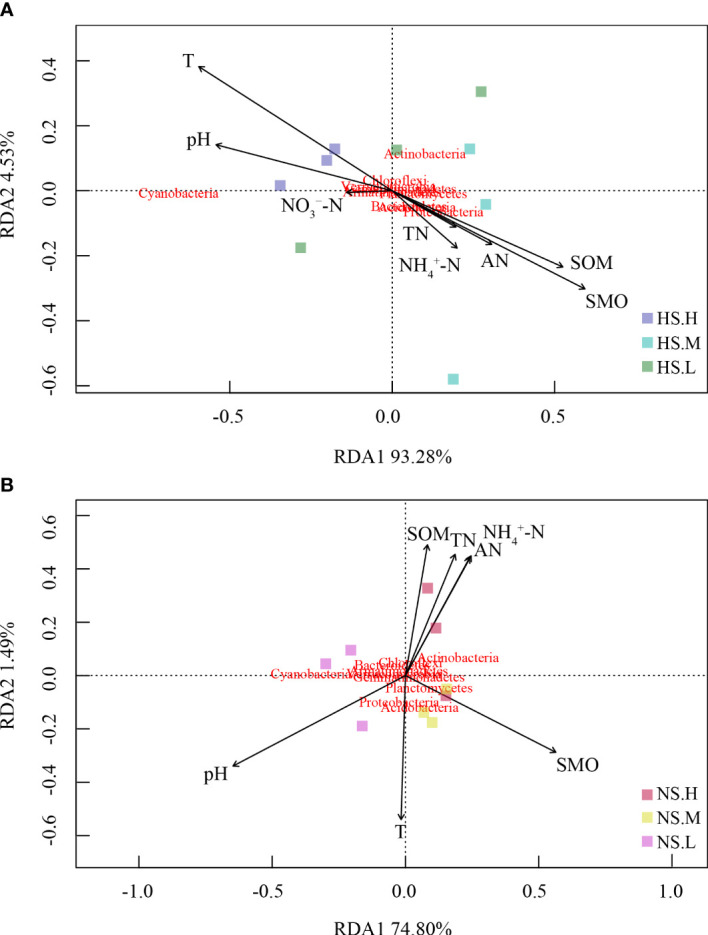
Redundancy analysis (RDA) demonstrating the correlations of bacterial communities to a series of environmental factors. Snow-cover **(A)**, Snow-uncover **(B)**. Total nitrogen (TN), Soil organic matters (SOM), Soil moisture (SMO), pH, Temperature (T), Available nitrogen (AN), Ammonium (NH_4_^+^-N), Nitrate (NO_3_^−^-N). NS.L (Snow-uncover and light nitrogen), HS.L (Snow-cover and light nitrogen), NS.M (Snow-uncover and moderate nitrogen), HS.M (Snow-cover and moderate nitrogen), NS.H(Snow-uncover and high nitrogen), HS.H (Snow-cover and high nitrogen). Different colors represent the different bacterial communities.

**Figure 8:**
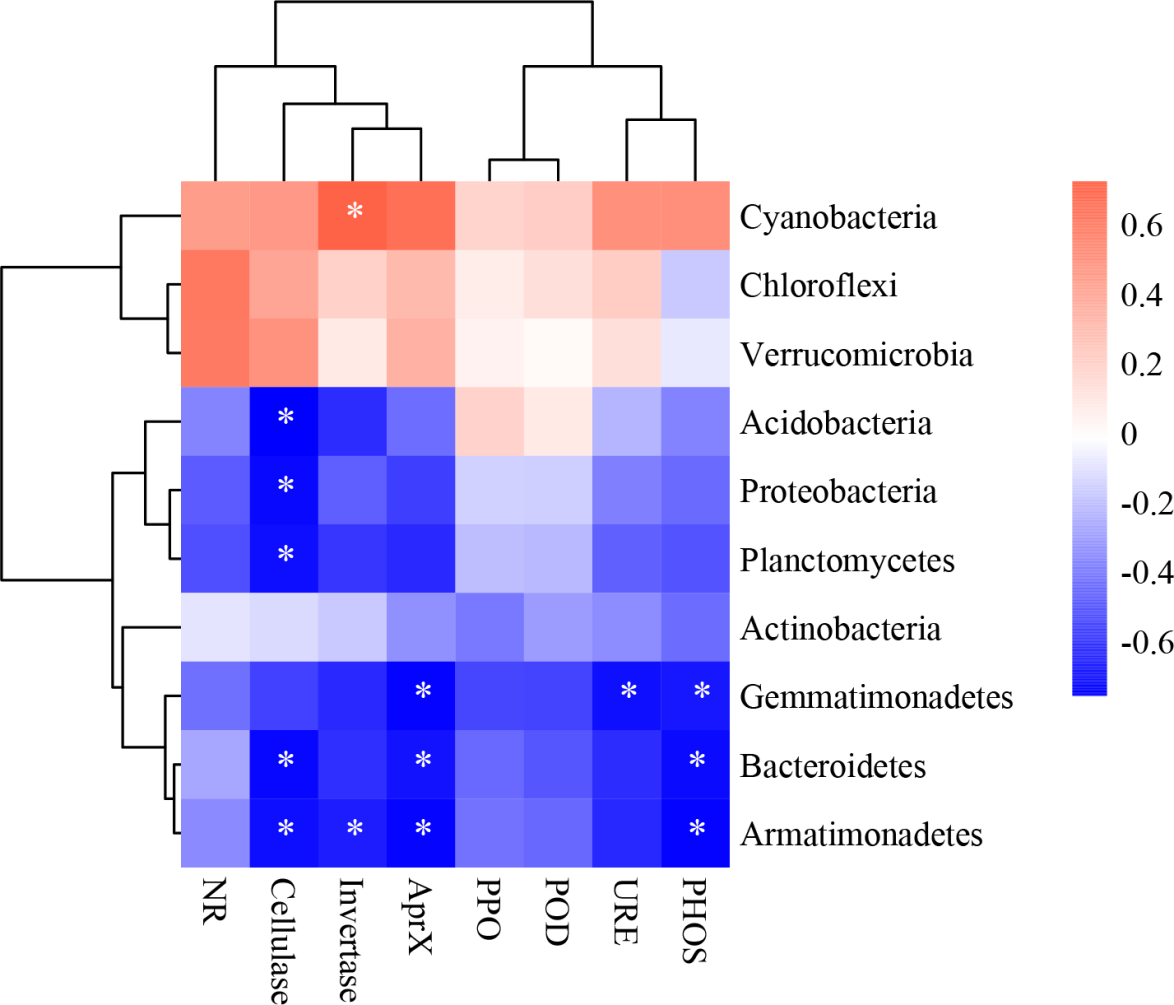
The correlation heatmap of dominant bacterial phyla and enzymes. *P < 0.05.

In **Figure 7**, for snow-cover, Axis 1 explained 93.28% and Axis 2 explained 4.53% of the total variance, whereas, for snow-uncover, Axis 1 explained 74.8% and Axis 2 explained 1.49% of the total variance. The most significant feature between environmental factors and bacterial abundance with snow-cover was that TN, AN, SOM, and NH_4_^+^-N were highly negatively correlated when high N was added, whereas bacterial abundance with snow-uncover was highly positively correlated when moderate N was added, indicating that snow-cover altered the role of bacteria in nutrient cycling under different N addition levels. In addition, pH and temperature were negatively correlated with the above factors but positively correlated with cyanobacteria abundance. Interestingly, SMO and temperature were negatively correlated with snow-cover and were positively correlated with snow-uncover. Moreover, cyanobacteria, chloroflexi and verrucomicrobia were positively correlated with enzymes, whereas the other phyla were negatively correlated with enzymes (**Figure 8**). For instance, cellulase showed a negative correlation with acidobacteria, proteobacteria, planctomycetes, bacteroidetes and armatimonadetes.

**Figure 9:**
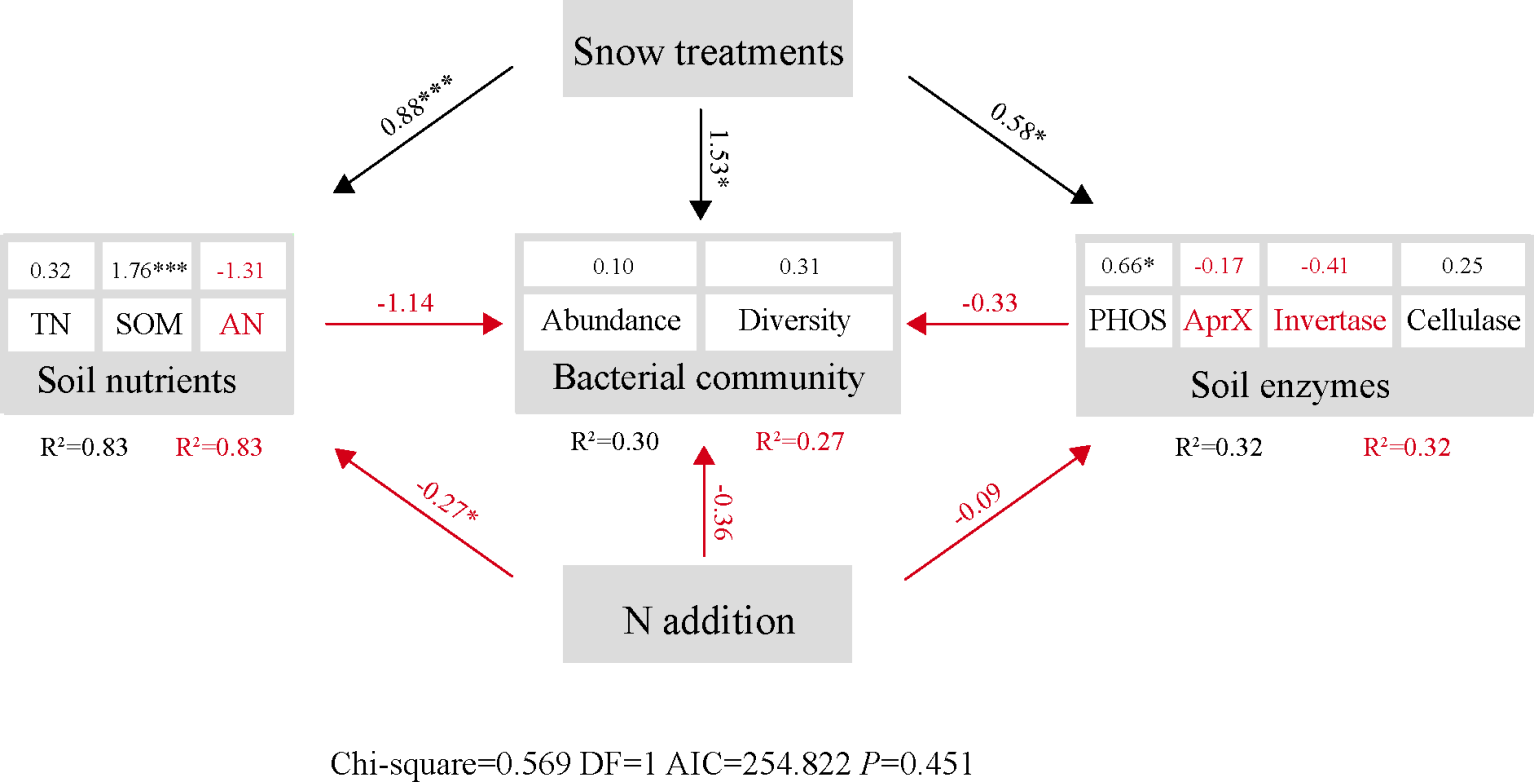
Structure equation model (SEM) analysis of nitrogen addition and snow-cover treatments on soil bacterial abundance (OTUs), diversity (1-Simpson) and soil enzymes *via* pathways of soil nutrients. The soil nutrient, enzymes, and bacterial community were divided into compositive variables. Numbers adjacent to measured variables are their coefficients with composite variables. Numbers show the path coefficients. Red and black indicates negative and positive relationships, respectively. The red R^2^ indicate the marginal R^2^. The black R^2^ indicate the conditional R^2^. *P < 0.05, ***P < 0.001.

Overall, we characterized the soil nutrients, enzyme activity, and bacterial community across various N addition under different snow-cover treatments by SEM analysis (Chi-square=0.569; DF=1; AIC=254.822; *P*=0.451). Snow-cover treatments were positively correlated with soil nutrients (*P* < 0.001), enzyme activities (*P* < 0.05), and bacterial communities (*P* < 0.05), while N addition was negatively correlated with them (**Figure 9**). The results also showed that soil nutrients (*P* < 0.05) and enzyme activity were negatively linked with bacterial communities. Additionally, an examination of the path coefficients revealed that snow-cover treatments altered the bacterial communities directly, whereas N addition changed the bacterial community indirectly by changing soil nutrients. Particularly, SOM (*P* < 0.001) and PHOS (*P* < 0.05) made significant contributions to the model, respectively.

## 4 Discussion

### 4.1 Snow-uncover altered the nutrient status of BSCs under different nitrogen levels

Snow-cover removal reduced the accumulation of soil nutrients, which weakened the responses of soil nutrients to the addition of N (**Table 1**). The significance test concluded that snow-uncover significantly reduced the TN, SOM, NH_4_^+^-N, and AN content under light and moderate N additions. This may be because of the absence of snow-cover, which caused low SMO to inhibit the biological activity of BSCs (Zhao et al., 2018). The presence of snow protects BSCs from perturbation by freeze-thaw cycles, enhances microbial and heterotrophic activity, active soil N mineralization, and retains relatively high levels of N nutrients (Freppaz et al., 2012), facilitating nutrient sequestration by BSCs (Thomas and Dougill, 2006). Whereas the removal of snow-cover accelerates the physical disintegration and loss of nutrients from bare soil and damages microbial cell structure (Hui et al., 2022). Moreover, it reduces the concentration of AN, inhibits microbial metabolism, and alters the availability of soil nutrients, which is detrimental to the growth and development of BSCs (Hui et al., 2022). At the same time, the content of soil nutrients under snow-uncover decreased with N addition and significantly increased with high N addition, which may be due to the fact that N fertilization increased soil N availability in N-limited desert ecosystems (Fan et al., 2013). According to Yan et al. (2018), the addition of N caused a decrease in pH and an increase in inorganic N, which significantly reduced bacterial abundance. Additionally, soil acidification led to an increase in acidophilic bacteria, which significantly reduced bacterial diversity (Rousk et al., 2010; Yan et al., 2018). Knorr et al. (2005) concluded that N addition accelerated decomposition mostly at the beginning but prevented it later on. This might be due to the demand for less soluble carbon substrates for microbial decomposition in the early stages (Berg and Matzner, 1997; Carreiro et al., 2000), while N saturation occurs in the later stages. In general, soil nutrients were more sensitive to snow-cover treatment than to N (**Figure 9**). N-induced soil acidification affects bacterial communities, and snow-cover can suppress these effects by increasing soil pH and enhancing soil N availability (Zhang et al., 2015; She et al., 2018). Indeed, microbial metabolism and nutrient cycling may be inhibited by hypoxia and humidity at excessive snow depths (Edwards and Jefferies, 2013; Zhao et al., 2018), which also deserves further investigation in the future.

### 4.2 Influence of snow-cover on enzyme activities under different N addition levels

Most soil enzymes in the winter BSCs were significantly correlated with N addition, and snow-cover removal attenuated this correlation (**Table 2**). The ANOVA analysis indicated that the enzyme activities of URE, Invertase, PPO, POD, and AprX were significantly correlated with N addition. URE (*P* < 0.001), Invertase (*P* < 0.01), and AprX (*P* < 0.01) as typical hydrolytic enzymes showed high sensitivity to N addition (Miralles et al., 2021), which suggests that soil enzymes have adapted to survive drought stress in arid areas, making them more nutrient-dependent (Ren et al., 2020). POD and PPO as oxidative enzymes increased significantly with N addition, perhaps this is because they are produced by fungi (Wang et al., 2022), which have a high tolerance for acidity and are sensitive to N availability (Talbot et al., 2015). Previous research has demonstrated that after the removal of the snow-cover, frequent freeze-thaw cycles caused the death of soil microorganisms, which resulted in the temporary release of enzymes and nutrients. This fundamentally reduced the source of soil enzymes and constrained enzyme activity (Wu, 2020).

Our analysis indicated that snow-cover removal weakened the response of soil enzymes to N addition, and this effect differed significantly with N addition (**Figure 2**). This might be owing to the harsh environment of low temperature and SMO after snow-cover removal, which caused reduced resource availability and microbial mortality (Zhou et al, 2012; Xiao et al., 2018), or it could be due to the fact that snow-cover removal changed the responsiveness of soil pH to N addition, affecting enzyme activity under different N additions (Carrara et al., 2018). After snow-cover removal, the addition of N significantly enhanced enzyme activities with light N addition, and further N addition had no significant influence. Carrara et al. (2018) identified the reason for the stagnation in enzyme activity: excess N led to a decrease in subsurface carbon allocation, which changed the composition of the bacterial community and inhibited enzyme activities. Likewise, there was a significant reduction in invertase at high N addition, which may also be related to the carbon-limiting condition (Peng et al., 2019). However, PPO activity was significantly reduced under high N addition because excess N inhibited its secretion (Liu et al., 2020b). In brief, N is the main limiting factor for soil enzymes in desert areas, and the loss of snow-cover results in less enhanced enzyme activities to N addition.

### 4.3 Effects of snow-cover on bacterial structure and function under different N addition levels

Snow-cover removal altered the bacterial abundance and diversity, with differences being significant for either high or light N additions (**Figure 2**). Bacterial diversity was highest under light N additions with snow-uncover and decreased significantly with N addition. A similar finding was reached by earlier studies in arid and semi-arid terrestrial ecosystems: bacterial diversity declines linearly with increased N addition (Wang et al., 2015; Qi et al., 2021). The majority of desert bacteria may be better suited to alkaline soil under light N addition (Qi et al., 2021), whereas high N addition results in soil acidification, which diminishes bacterial diversity and reduces BSCs resistance to the disturbance in the winter (Wang et al., 2015). After the removal of the snow, increasing N addition caused the soil pH to decreased significantly, which led to changes in bacterial diversity. Meanwhile, the abundance of bacteria under snow-cover was significantly lowest at high N addition. There could be a decreased carbon allocation of belowground as a result of the increasing N addition, which inhibited bacterial abundance (Wang et al., 2021b). Our results also suggest that the snow-covered bacterial community showed more positive correlations and synergistic relationships, and the complex network revealed that snow-cover promoted the stability of the bacterial community and provided better ecological services. In contrast, more sporadic and more negative connections were observed in the snow-uncovered bacterial community, revealing more competition and a fragile community. The ground is covered by snow throughout the winter, which helps to keep the soil at an appropriate temperature for microbial community growth and development (Wan et al., 2020). Snow-cover may also be the primary factor driving bacterial networks (Wan et al., 2020).

**Figure 10:**
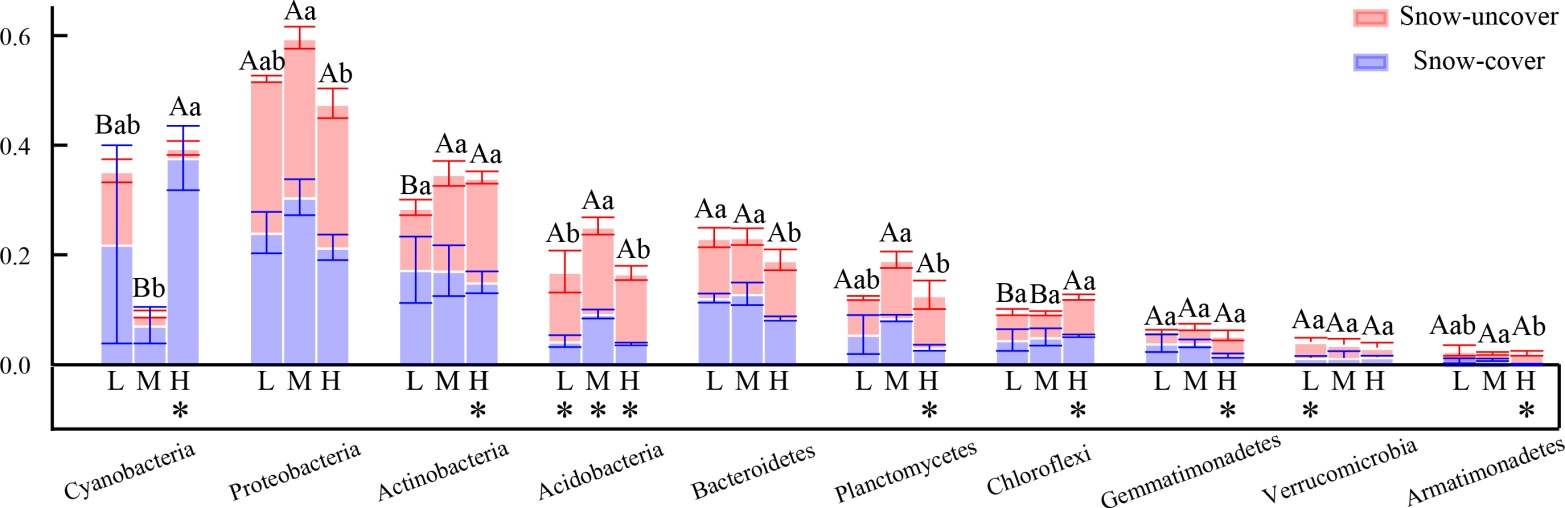
Relative abundance (%) of bacterial composition at the phylum level. L = 1.8 g N·m^−2^·year^−1^, M = 3.6 g N·m^−2^·year^−1^, H = 7.2 g N·m^−2^·year^−1^. Different lowercase and capital letters denote significant differences between treatments of snow-cover and snow-uncover treatments, respectively. Error bars represent the standard deviation (SD) (n = 3). *P < 0.05.

Our results in the present study revealed that cyanobacteria, proteobacteria, and actinobacteria were the dominant bacterial phyla in winter (**Figures 4**, **10**), which is similar to that of Huang et al. (2021). Proteobacteria, which initially dominated the snow-covered bacterial community, gave way to cyanobacteria when sufficient N was added, creating a species-diverse but low-abundance community (**Figure 4**). The change in the status of the cyanobacteria and proteobacteria in the bacterial community is due to their different forms of nutrient utilization. When sufficient N was added, the proteobacteria that were initially dominant in the snow-covered bacterial community gave way to cyanobacteria, forming a species-diverse but low-abundance population (**Figure 4**). Various bacteria and soil enzymes accelerated the decomposition of organic N and promoted the formation of inorganic N with the continuous addition of N, forming a bacterial community dominated by proteobacteria (Zhao et al., 2016b). Subsequently, with snow-cover and high N addition, cyanobacteria could fix molecular N and recruit more bacteria and enzymes to grab the niche of proteobacteria, and the BSCs entered the algal stage (Zhou et al., 2020; Zhang et al., 2021b). A low abundance of bacterial community caused a slow rate of breakdown and increased nutrient accumulation (TN, SOM, AN, NH_4_^+^-N, and NO_3_^−^-N), which also coincidentally provided nutrients for soil enzymes. (**Figure 9**). In fact, proteobacteria continued to remain the most advantageous of the snow-uncovered phyla, with cyanobacteria trailing behind. However, the snow-uncovered community performed significant glycan, amino acid, carbohydrate and lipid metabolism.

### 4.4 Internal mechanism drives soil nutrients and microbial community

We explored the pathways of winter BSCs and elaborated on the effect of snow-cover treatments on soil nutrients, enzymes and bacterial communities with different N addition. SEM analysis demonstrated that the higher SMO and suitable microenvironment created by the snow-cover accumulated soil nutrients and activated the bacterial communities (**Figure S1**). The effectiveness of soil enzymes and bacterial communities in utilizing nutrients was further increased with an increase in N addition, which led to a linear reduction in soil nutrients (Xie et al., 2022). The insignificant effect of soil enzymes may be due to the fact that bacteria produce extracellular enzymes to meet their nutrient and energy needs in nutrient-limited environments, which break down aggregated organic matter into small molecules (Ullah et al., 2019). It is also possible that BSCs enzyme activity in winter is influenced by specific functional bacterial communities rather than the entire bacterial communities (Ling et al., 2014).

Correspondingly, due to the absence of snow-cover, the BSCs were consequently unable to retain nutrients during the winter and had to rely on the addition of external N. Low SMO and limited soil nutrients hindered bacterial degradation of SOM (**Figure S1**). Thus, N addition has an indirect effect on the bacterial community by altering soil nutrients.

## 5 Conclusion

The results revealed that removing snow-cover decreased the concentration of nutrients stored in the soil (TN, SOM, AN, NH_4_^+^-N, and NO_3_^−^-N), inhibited enzymes, and suppressed bacterial community. Snow-cover removal also attenuated the response of soil nutrients and enzyme activity to N addition, indicating a weakened response of the desert ecosystems to N deposition and may be detrimental to the stability of desert ecosystems and the development of BSCs. These findings will provide a theoretical basis and direction for the application of BSCs in ecological restoration. In addition, our study complements the study on the correction of winter snowfall on the different N deposition, enhances the parameters of the nutrient cycle prediction model, and underlines the significance of snow-cover in arid regions.

## Data availability statement

The original contributions presented in the study are publicly available. This data can be found here: https://www.ncbi.nlm.nih.gov/bioproject/PRJNA944592.

## Author contributions

YY analyzed data and wrote the manuscript. WL designed the experiment and provided financial support. JA developed the original idea and modified the manuscript. BS did the literature search.
