# Supplementary material for: Snow-cover loss attenuates the effects of N addition on desert nutrient cycling and microbial community
Source: Front Plant Sci. 2023 Jul 20;14:1166897. doi: 10.3389/fpls.2023.1166897 (PMC10400093; doi:10.3389/fpls.2023.1166897)
Supplement: Supplementary file 1 [file DataSheet_1.pdf]

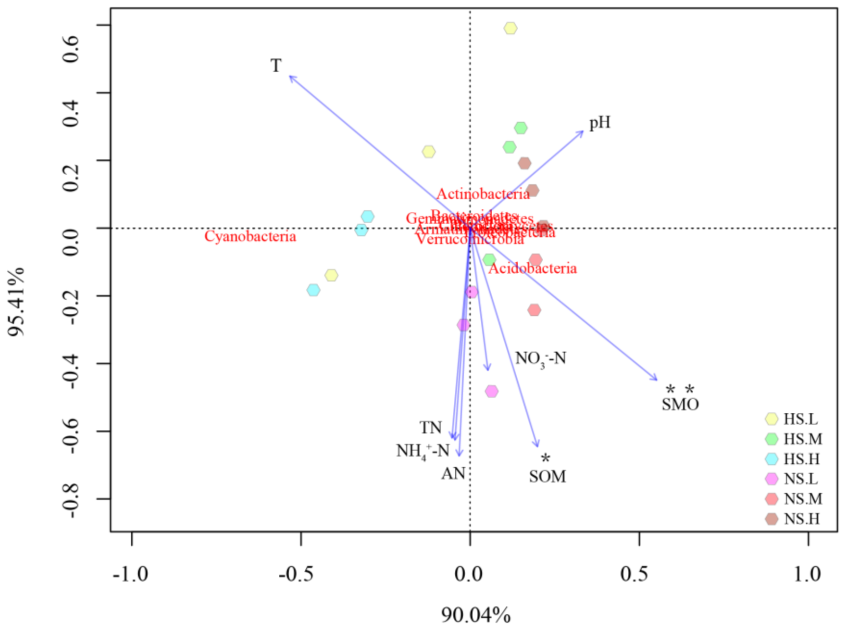

FIGURE S1

Redundancy analysis (RDA) demonstrating the correlations of bacterial communities to a series of environmental factors. Total nitrogen (TN), soil organic matters (SOM), soil moisture (SMO), pH, temperature (T), available nitrogen (AN), ammonium (NH_4^+-N), nitrate (NO_3^--N). NS.L (Snow-uncover and light nitrogen), HS.L (Snow-cover and light nitrogen), NS.M(Snow-uncover and moderate nitrogen), HS.M (Snow-cover and moderate nitrogen), NS.H(Snow-uncover and high nitrogen), HS.H (Snow-cover and high nitrogen). Different colors represent the different bacterial communities. * $P < 0.05$, ** $P < 0.01$, *** $P < 0.001$.

Table S1 Effects of snow-cover and nitrogen applications on bacterial abundance. F-value (*P*-value). L = 1.8 g N·m^{-2}·year^{-1}, M = 3.6 g N·m^{-2}·year^{-1}, H = 7.2 g N·m^{-2}·year^{-1}. Snow-cover (S); snow-uncover (Uns).

Phylum	L	M	H	S	Uns
Cyanobacteria	1.08 (0.36)	3.39 (0.14)	38.83 (0.00)	4.91 (0.05)	47.44 (0.00)
Proteobacteria	0.19 (0.69)	0.11 (0.76)	4.99 (0.09)	1.51 (0.29)	3.15 (0.12)
Actinobacteria	3.46 (0.14)	4.17 (0.11)	12.85 (0.02)	0.44 (0.66)	42.53 (0.00)
Acidobacteria	11.32 (0.03)	14.57 (0.02)	81.71 (0.00)	8.52 (0.02)	4.60 (0.06)
Bacteroidetes	6.99 (0.06)	0.26 (0.636)	2.28 (0.21)	2.41 (0.17)	0.49 (0.63)
Planctomycetes	0.036 (0.86)	4.15 (0.11)	19.05 (0.01)	2.47 (0.16)	6.81 (0.03)
Chloroflexi	0.10 (0.76)	0.00 (1.00)	10.35 (0.03)	0.25 (0.79)	16.61 (0.00)
Gemmatimonadetes	3.91 (0.12)	0.67 (0.46)	14.11 (0.02)	3.18 (0.11)	5.36 (0.05)
Verrucomicrobia	8.86 (0.04)	3.15 (0.15)	0.51 (0.52)	0.23 (0.80)	0.74 (0.52)
Armatimonadetes	0.34 (0.59)	0.20 (0.68)	40.22 (0.00)	3.09 (0.12)	1.43 (0.31)
